# Supplementary material for: 3D-Printed EVA Devices for Antiviral Delivery and Herpes Virus Control in Genital Infection
Source: Viruses. 2022 Nov 11;14(11):2501. doi: 10.3390/v14112501 (PMC9696101; doi:10.3390/v14112501)
Supplement: Supplementary file 1 [file viruses-14-02501-s001.zip › viruses-1935995-SI.pdf]

3D-Printed EVA Devices for Antiviral Delivery and Herpes Virus Control in Genital Infection

Victor de Carvalho Rodrigues [1], Iara Zanella Guterres [2], Beatriz Pereira Savi [2], Izabella Thaís Silva [2,3], Gislaine Fongaro [2,*] and Gean Vitor Salmoria [1,4,*]

1 Nimma, Department of Mechanical Engineering, Federal University of Santa Catarina, Florianópolis 88040-900, SC, Brazil
2 Laboratory of Applied Virology, Department of Microbiology, Imunology and Parasitology, Federal University of Santa Catarina, Florianópolis 88040-900, SC, Brazil
3 Laboratory of Pharmacognosy, Department of Pharmaceutical Sciences, Federal University of Santa Catarina, Florianópolis 88040-900, SC, Brazil
4 Biomechanics Engineering Laboratory, University Hospital (HU), Federal University of Santa Catarina, Florianópolis 88040-900, SC, Brazil
* Correspondence: gislaine.fongaro@ufsc.br (G.F.); gean.salmoria@ufsc.br (G.V.S.)

Supplementary Figures:

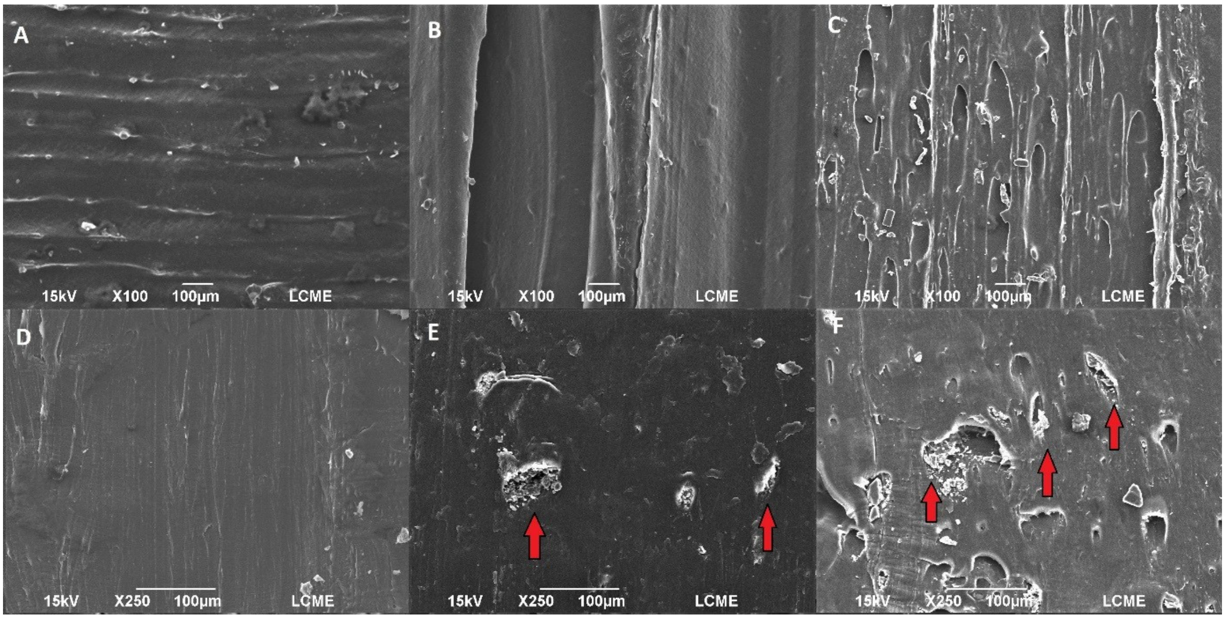

Figure S1. SEM images of surface (top) and cross-section (bottom) of 3d printed IUD of EVA-P (A,D), EVA-P10 (B,E) and EVA-P20 (C,F). Red arrows indicate regions with drug particles, confirming the incorporation of acyclovir into the EVA matrix.
